# 自体造血干细胞移植治疗初治多发性骨髓瘤的临床分析

**DOI:** 10.3760/cma.j.issn.0253-2727.2021.05.007

**Published:** 2021-05

**Authors:** 传营 耿, 光忠 杨, 国蓉 王, 慧娟 王, 慧星 周, 之尧 张, 原 菅, 文明 陈

**Affiliations:** 首都医科大学附属北京朝阳医院血液科，北京 100020 Department of Hematology, Beijing Chaoyang Hospital, Capital Medical University, Beijing 100020, China

**Keywords:** 多发性骨髓瘤, 自体造血干细胞移植, 生存, 预后因素, Multiple myeloma, Autologous stem cell transplantation, Survival, Prognostic factors

## Abstract

**目的:**

评估自体造血干细胞移植（auto-HSCT）对初治多发性骨髓瘤（MM）疗效及生存的影响。

**方法:**

回顾性分析2008年10月1日至2019年10月1日243例65岁以下接受auto-HSCT的初治MM患者，同时以同期176例≤65岁适合移植但未进行auto-HSCT的初治MM患者作为对照，评估auto-HSCT对患者疗效及生存的影响。为平衡auto-HSCT和非auto-HSCT患者之间各因素的分布，利用倾向性评分匹配技术按照1∶1比例匹配以减少组间的偏差。

**结果:**

通过倾向性评分匹配分析，共筛选出128例患者（每组64例）。64例患者诱导治疗后接受auto-HSCT，24例（37.5％）获得严格意义的完全缓解（sCR），16例（25.0％）获得完全缓解（CR），15例（23.4％）获得非常好的部分缓解（VGPR），9例（14.1％）获得部分缓解（PR），auto-HSCT组疗效明显优于非auto-HSCT组（*P*＝0.032）。与非auto-HSCT组相比，auto-HSCT组总生存（OS）和无进展生存（PFS）期明显延长［OS：87.6（95％ *CI* 57.3～117.9）个月对53.9（95％ *CI* 36.1～71.7）个月，*P*＝0.011；PFS：42.2（95％ *CI* 29.9～54.5）个月对22.4（95％ *CI* 17.1～27.7）个月，*P*＝0.007］。多因素分析显示auto-HSCT是OS（*HR*＝0.448，95％*CI* 0.260～0.771，*P*＝0.004）和PFS（*HR*＝0.446，95％*CI* 0.280～0.778，*P*＝0.003）的独立保护因素。

**结论:**

auto-HSCT可改善适合移植初治MM患者的OS和PFS。

## Abstract (en)

**Objective:**

To evaluate the effect of autologous stem cell transplantation（auto-HSCT）on treatment remission and survival of newly diagnosed multiple myeloma（MM）patients.

**Methods:**

A total of 243 new diagnosed MM patients（age ≤65 years）who had received auto-HSCT were selected, and 176 MM patients（age ≤65 years）who had not received auto-HSCT were selected as the control group to evaluate the effect of auto-HSCT on the remission and survival. To balance the distribution of prognostic factors between auto-HSCT and non-auto-HSCT patients, the propensity score matching technique was used to reduce the bias between groups in a 1∶1 scale, 64 in each group, and correlation analysis was performed.

**Results:**

A total of 128 patients（64 cases in each group）were screened by propensity score matching analysis. 64 patients received auto-HSCT after induction therapy. After auto-HSCT, 24 patients （37.5％）obtained sCR, 16 patients（25.0％）obtained CR, 15 patients（23.4％）obtained VGPR, and 9 patients（14.1％）obtained PR. The efficacy of patients with auto-HSCT was significantly better than that of non-auto-HSCT patients（*P*＝0.032）. Progression-free survival（PFS）and overall survival（OS）were significantly longer in auto-HSCT patients compared with non-auto-HSCT patients［PFS: 42.2（95％ *CI* 29.9–54.5）months *vs* 22.4（95％ *CI* 17.1–27.7）months, *P*＝0.007; OS: 87.6（95％ *CI* 57.3–117.9） months *vs* 53.9（95％ *CI* 36.1–71.7）months, *P*＝0.011］. Multivariate analysis confirmed that auto-HSCT had a favorable effect on OS（*HR*＝0.448, 95％*CI* 0.260–0.771, *P*＝0.004）and PFS（*HR*＝0.446, 95％*CI* 0.280–0.778, *P*＝0.003）.

**Conclusion:**

These results demonstrated that auto-HSCT was a favorable prognostic factor for newly diagnosed MM patients.

自体造血干细胞移植（auto-HSCT）明显改善了适合移植多发性骨髓瘤（MM）患者的预后，使MM治疗取得巨大进步[1]。传统药物时代，auto-HSCT可明显改善适合移植的MM患者无进展生存（PFS）和总生存（OS），大剂量马法兰联合auto-HSCT是适合移植的初治MM患者标准一线治疗方案[2]–[4]。然而，auto-HSCT虽然可延长患者PFS期，但OS无获益，随着硼替佐米、来那度胺和地塞米松（VRD）诱导方案在MM治疗中的应用，MM患者疗效得到进一步改善[5]。但在中国，初治MM患者应用硼替佐米、来那度胺等两种新药联合诱导治疗的比例不高，移植仍占据重要地位，基于硼替佐米和（或）免疫调节剂（IMiD）诱导治疗下的auto-HSCT疗效有待进一步评价。本研究中，我们回顾性分析419例随访超过1年的在首都医科大学北京朝阳医院进行治疗的新诊断MM患者，评估新药时代auto-HSCT对适合移植的初治MM患者疗效及生存的影响。

## 病例与方法

一、临床资料

回顾性分析2008年10月1日至2019年10月1日在我院长期治疗随访进行auto-HSCT的243例65岁以下初治MM患者，同时以同期≤65岁适合移植但未进行auto-HSCT的176例初治MM患者为对照，诊断及分期参照国际骨髓瘤工作组标准[6]。进行auto-HSCT的患者在开始诱导治疗后的12个月（中位5个月）内接受auto-HSCT。收集患者初次治疗前骨髓标本，采用CD138^+^磁珠富集细胞的荧光原位杂交（FISH）检测包括1q21扩增、del（17p13）、t（14;16）、t（4;14）和t（11;14）在内的细胞遗传学异常。主要收集患者年龄、性别、细胞遗传学资料等。所有患者均无严重心、肺、肝、肾等脏器并发症。

二、治疗

患者诊断后均接受至少含有1种新药（硼替佐米、沙利度胺、来那度胺）的诱导治疗，主要包括硼替佐米为基础、IMiD为基础和硼替佐米联合IMiD治疗方案。硼替佐米为基础的治疗方案主要包括BD（硼替佐米+地塞米松）、BCD（硼替佐米+环磷酰胺+地塞米松）、BAD（硼替佐米+阿霉素+地塞米松）等；IMiD为基础治疗方案包括LD（来那度胺+地塞米松）、CTD（环磷酰胺+沙利度胺+地塞米松）等；硼替佐米联合IMiD治疗方案包括BTD（硼替佐米+沙利度胺+地塞米松）、BLD（硼替佐米+来那度胺+地塞米松）、BCDT（硼替佐米+环磷酰胺+地塞米松+沙利度胺）、BADT（硼替佐米+阿霉素+地塞米松+沙利度胺）和BRCD（硼替佐米+来那度胺+环磷酰胺+地塞米松）等。患者诱导治疗后接受环磷酰胺（2～3 g·m^−2^·d^−1^）动员，应用环磷酰胺第7天应用G-CSF 5～10 µg·kg^−1^·d^−1^，直至干细胞采集结束。在回输造血干细胞前，按照200 mg/m^2^的剂量应用美法仑一次进行预处理，第3天回输自体造血干细胞，待粒细胞和血小板重建后确定移植成功。auto-HSCT 3个月后进行疗效评估，如患者未获得非常好的部分缓解（VGPR）及以上疗效，再接受2个诱导治疗方案进行巩固治疗。患者随后接受硼替佐米、来那度胺或沙利度胺进行维持治疗，直至疾病进展（PD）。

三、随访及疗效判定标准

采用查阅门诊/住院病历和电话随访方式获得患者生存资料。根据IMWG标准[7]评估患者疗效，包括严格意义的完全缓解（sCR）、完全缓解（CR）、VGPR、部分缓解（PR）、疾病稳定（SD）、PD。主要观察指标为PFS和OS。PFS时间定义为从诊断到疾病进展或死亡的时间，OS时间定义为从诊断到死亡或末次随访的时间。无法随访的患者将最后一次随访日期作为删失。

四、统计学处理

采用SPSS 22.0及R 2.15.2软件进行统计学分析。卡方检验用来检验分类变量。通过Kaplan-Meier法进行生存分析，并采用Log-rank检验比较组间差异。Cox比例风险回归分析用于评估预后影响因素。为平衡auto-HSCT和非auto-HSCT患者之间各因素的分布，利用倾向性评分匹配技术平衡auto-HSCT和非auto-HSCT组患者年龄、国际评分系统（ISS）分期、血红蛋白、血肌酐、校正血清钙、乳酸脱氢酶、del（17p13）、t（14;16）、t（4;14）和诱导治疗方案，按1∶1进行匹配，设卡钳值为0.10。*P*<0.05为差异有统计学意义。

## 结果

一、患者基线特征

全部419例MM患者的临床特点见表1。中位年龄为55（29～65）岁，男女比为1.13（223/196），IgG型（46.1％）MM最常见，181例（43.9％）为ISS Ⅲ期；所有患者均应用含硼替佐米或沙利度胺或来那度胺等新药的方案进行诱导治疗，其中205例（49.4％）接受硼替佐米基础方案治疗，57例（13.7％）接受IMiD基础方案治疗，153例（36.9％）接受硼替佐米联合IMiD方案治疗。诱导治疗后，243例患者接受auto-HSCT治疗。auto-HSCT患者与非auto-HSCT患者在年龄、ISS分期、血红蛋白、血肌酐、del（17p13）等方面差异有统计学意义（表1）。为降低组间因素对移植疗效的影响，应用倾向性评分匹配auto-HSCT组与非auto-HSCT组，匹配后各因素组间差异均无统计学意义。

**表1:** 倾向性评分（PSM）匹配前后auto-HSCT和非auto-HSCT组多发性骨髓瘤（MM）患者基线特征

临床特征	总体（419例）	PSM前			PSM后		
		auto-HSCT组（243例）	非auto-HSCT组（176例）	*P*值	auto-HSCT组（64例）	非auto-HSCT组（64例）	*P*值
性别［例（％）］				0.792			0.111
男	223（53.2）	128（52.7）	95（54.0）		29（45.3）	38（59.4）	
女	196（46.8）	115（47.3）	81（46.0）		35（54.7）	26（40.6）	
中位年龄（岁）	55（29～65）	54（29～65）	56（38～65）	<0.001	56（38～65）	55（42～65）	0.522
MM类型［例（％）］				0.146			0.135
IgG	193（46.1）	110（45.3）	83（47.2）		28（43.8）	32（50.0）	
IgA	83（19.8）	47（19.3）	36（20.5）		11（17.2）	15（23.4）	
IgD	27（6.4）	22（9.1）	5（2.8）		6（9.4）	1（1.6）	
轻链型	102（24.3）	57（23.5）	45（25.6）		17（26.6）	11（17.2）	
非分泌型	14（3.3）	7（2.9）	7（4.0）		2（3.1）	5（7.8）	
ISS分期［例（％）］				<0.001			0.349
Ⅰ期	88（21.4）	67（28.0）	21（12.1）		11（17.2）	15（23.4）	
Ⅱ期	143（34.7）	80（33.5）	63（36.4）		32（50.0）	24（37.5）	
Ⅲ期	181（43.9）	92（38.5）	89（51.4）		21（32.8）	25（39.1）	
血红蛋白［例（％）］				0.028			0.588
<100g/L	151（66.5）	49（57.6）	102（71.8）		40（62.5）	37（57.8）	
≥100g/L	76（33.5）	36（42.4）	40（28.2）		24（37.5）	27（42.2）	
血肌酐［例（％）］				0.038			0.544
≤177µmol/L	179（82.9）	71（89.9）	108（78.8）		57（89.1）	59（92.2）	
>177µmol/L	37（17.1）	8（10.1）	29（21.2）		7（10.9）	5（7.8）	
校正血清钙［例（％）］				0.599			0.795
≤2.75mmol/L	180（88.2）	70（89.7）	110（87.3）		56（87.5）	55（85.9）	
>2.75mmol/L	24（11.8）	8（10.3）	16（12.7）		8（12.5）	9（14.1）	
乳酸脱氢酶［例（％）］				0.889			0.811
≤250U/L	171（85.1）	65（85.5）	106（84.8）		54（84.4）	53（82.8）	
>250U/L	30（14.9）	11（14.5）	19（15.2）		10（15.6）	11（17.2）	
FISH检测细胞学异常［例（％）］							
del（17p13）				0.048			0.752
异常	21（9.9）	16（13.4）	5（5.3）		6（9.4）	5（7.8）	
无异常	192（90.1）	103（86.6）	89（94.7）		58（90.6）	59（92.2）	
t（14;16）				0.682			0.402
异常	10（4.7）	5（4.1）	5（5.3）		4（6.3）	2（3.1）	
无异常	205（95.3）	116（95.9）	89（94.7）		60（93.8）	62（96.9）	
t（4;14）				0.114			0.504
异常	45（20.9）	30（24.8）	15（16.0）		14（21.9）	11（17.2）	
无异常	170（79.1）	91（75.2）	79（84.0）		50（78.1）	53（82.8）	
诱导治疗方案［例（％）］				0.120			0.520
硼替佐米基础	205（49.4）	124（51.5）	81（46.6）		27（42.2）	26（40.6）	
免疫调节剂基础	57（13.7）	26（10.8）	31（17.8）		5（7.8）	9（14.1）	
硼替佐米和免疫调节剂基础	153（36.9）	91（37.8）	62（35.6）		32（50.0）	29（45.3）	

注：allo-HSCT：异基因造血干细胞移植

二、疗效分析

auto-HSCT组患者移植前接受中位5个疗程治疗，移植后接受中位2个疗程巩固治疗；非auto-HSCT患者接受中位9个诱导治疗。评估128例匹配后初治MM患者最佳疗效，37例（28.9％）患者获得sCR，31例（24.2％）获得CR，29例（22.7％）获得VGPR，31例（24.2％）获得PR。64例患者诱导治疗后接受auto-HSCT，auto-HSCT后，24例（37.5％）获得sCR，16例（25.0％）获得CR，15例（23.4％）获得VGPR，9例（14.1％）获得PR。非auto-HSCT患者中，13例（20.3％）获得sCR，15例（23.4％）获得CR，14例（21.9％）获得VGPR，22例（34.4％）获得PR，非auto-HSCT组缓解率明显低于auto-HSCT组（*P*＝0.032）。

三、生存分析

中位随访为32.9（1.3～139.2）个月。128例匹配后患者中，auto-HSCT和非auto-HSCT组患者中位OS期分别为87.6（95％ *CI* 57.3～117.9）个月和53.9（95％ *CI* 36.1～71.7）个月（*P*＝0.011）（图1A），中位PFS期分别为42.2（95％ *CI* 29.9～54.5）个月和22.4（95％ *CI* 17.1～27.7）个月（*P*＝0.007）（图1B）。auto-HSCT可明显延长MM患者PFS和OS期。

**图1:**
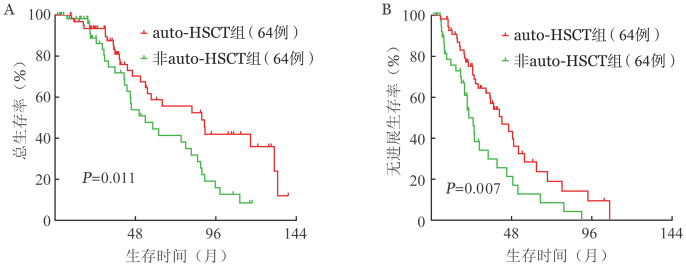
倾向性评分匹配后自体造血干细胞移植（auto-HSCT）患者和非auto-HSCT组患者总生存（A）和无进展生存（B）曲线

单因素Cox比例风险回归分析显示，校正血清钙>2.75 mmol/L、R-ISS Ⅲ期和auto-HSCT与患者OS相关（表2）。将年龄、HGB（<100 g/L，≥100 g/L）、血肌酐（≤177 µmol/L，>177 µmol/L）、校正血清钙（≤2.75 mmol/L，>2.75 mmol/L）、R-ISS分期（Ⅰ期，Ⅱ期，Ⅲ期）、诱导治疗方案（硼替佐米基础，IMiD基础，硼替佐米联合IMiD基础）和是否auto-HSCT纳入多因素分析，结果显示，年龄（*HR*＝0.959，95％*CI* 0.921～0.998，*P*＝0.038）、校正血清钙>2.75 mmol/L（*HR*＝2.438，95％ *CI* 1.209～4.917，*P*＝0.013）和auto-HSCT（*HR*＝0.448，95％*CI* 0.260～0.771，*P*＝0.004）是OS的独立影响因素，不同的诱导治疗方案与OS无明显相关性（表2）。在相同模型中对PFS进行多因素分析，发现auto-HSCT、年龄和HGB≥100 g/L是PFS的独立相关因素，auto-HSCT患者的PFS期明显延长（*HR*＝0.466，95％*CI* 0.280～0.778，*P*＝0.003），但不同的诱导治疗方案与PFS无明显相关性（表2）。

**表2:** 单因素与多因素Cox等比例回归分析新药诱导条件下多发性骨髓瘤患者总生存与无进展生存的影响因素

影响因素	总生存						无进展生存					
	单因素分析			多因素分析			单因素分析			多因素分析		
	*HR*	95％ *CI*	*P*值	*HR*	95％ *CI*	*P*值	*HR*	95％ *CI*	*P*值	*HR*	95％ *CI*	*P*值
年龄（岁）	0.965	0.925～1.006	0.095	0.959	0.921～0.998	0.038	0.941	0.901～0.983	0.006	0.943	0.904～0.983	0.006
HGB≥100g/L	0.707	0.412～1.214	0.209	0.799	0.433～1.475	0.473	0.574	0.328～1.007	0.053	0.504	0.287～0.886	0.017
血肌酐>177µmol/L	1.396	0.595～3.273	0.443	0.731	0.263～2.030	0.548	1.588	0.712～3.544	0.259	0.916	0.353～2.377	0.858
校正血清钙>2.75mmol/L	2.054	1.030～4.098	0.041	2.438	1.209～4.917	0.013	1.312	0.662～2.602	0.437	0.898	0.422～1.911	0.779
R-ISS分期												
Ⅰ期	1.000	ref		1.000	ref		1.000	ref		1.000	ref	
Ⅱ期	1.752	0.688～4.463	0.240	1.945	0.757～5.002	0.167	1.104	0.507～2.405	0.804	1.117	0.459～2.718	0.807
Ⅲ期	3.425	1.205～9.736	0.021	2.569	0.775～8.518	0.123	2.359	1.018～5.465	0.045	1.770	0.624～5.019	0.283
诱导治疗方案												
硼替佐米基础	1.000	ref		1.000	ref		1.000	ref		1.000	ref	
IMiD基础	1.161	0.503～2.678	0.727	0.953	0.392～2.314	0.915	2.090	0.883～4.942	0.093	2.413	1.011～5.762	0.047
硼替佐米联合 IMiD基础	1.208	0.653～2.234	0.547	1.092	0.576～2.073	0.787	1.211	0.701～2.092	0.492	1.306	0.752～2.268	0.343
auto-HSCT	0.503	0.294～0.860	0.012	0.448	0.260～0.771	0.004	0.503	0.303～0.835	0.008	0.466	0.280～0.778	0.003

注：R-ISS分期：修订的国际预后分期；IMiD：免疫调节剂；auto-HSCT：自体造血干细胞移植

## 讨论

与传统化疗相比，诱导治疗后进行auto-HSCT可进一步提高初治MM患者的缓解深度，延长患者PFS和OS时间，长期以来一直作为适合移植的初治MM患者的标准治疗方案。在EMN02/HO95的Ⅲ期临床试验中，614例适合auto-HSCT的初治MM患者接受VCD（硼替佐米+环磷酰胺+地塞米松）诱导治疗，诱导治疗后接受VMP（硼替佐米+美法仑+泼尼松）或auto-HSCT（单次或双次）巩固治疗，结果提示auto-HSCT可降低疾病进展和死亡风险[8]。Barlogie等[9]随机入组516例MM患者，分别接受auto-HSCT（261例）和标准剂量化疗（255例），两组获得PR及以上缓解率均为76％，长期随访（76个月）发现，auto-HSCT组患者7年PFS、OS率分别为17％、38％，标准剂量化疗组患者分别为14％、38％。IFM2009的一项随机Ⅲ期临床试验比较了RVD（来那度胺、硼替佐米、地塞米松）联合治疗与RVD+auto-HSCT治疗65岁以下初治MM患者的疗效，患者接受3个周期RVD诱导治疗后，分为继续使用5个周期RVD巩固治疗，或者接受auto-HSCT和2个周期RVD，之后所有患者均接受来那度胺维持治疗1年，结果提示，移植患者完全缓解率明显提高（59％对48％），MRD阴性率增加（79％对65％），中位PFS期延长（50个月对36个月），然而，移植患者OS无获益[5]。为此，新药时代MM造血干细胞移植的地位受到质疑。Koreth等[10]利用Meta分析了9个随机对照试验的2411例MM患者，结果发现auto-HSCT组患者死亡风险为0.92，进展风险为0.75，提示auto-HSCT可延长PFS期，但OS获益不明显。尽管一些研究对auto-HSCT在初治MM患者中的地位提出了质疑[11]，但研究结果认为auto-HSCT仍是经诱导治疗后适合移植患者的一线选择[12]–[14]。

本研究中，与auto-HSCT前相比，auto-HSCT后MM患者疗效进一步改善，而且，与非auto-HSCT的初治MM患者相比，auto-HSCT可明显改善患者缓解率。这表明auto-HSCT仍是初治MM患者诱导治疗后有效的巩固治疗方案。auto-HSCT、非auto-HSCT患者的中位OS时间分别为87.6（95％ *CI* 57.3～117.9）个月、53.9（95％ *CI* 36.1～71.7）个月（*P*＝0.011），中位PFS期分别为42.2（95％ *CI* 29.9～54.5）个月、22.4（95％ *CI* 17.1～27.7）个月（*P*＝0.007）。单因素分析显示，auto-HSCT可明显延长初治MM的PFS和OS，改善患者生存。多因素分析也同样表明auto-HSCT仍是初治MM患者PFS和OS的独立有利预后因素。因此，我们认为auto-HSCT对于适合移植的初治MM患者是一个良好的预后因素，在中国，auto-HSCT仍是适合移植患者的标准治疗。

本研究为回顾性、单中心研究，难免存在一定的局限性。而且，本研究的中位随访时间为32.9个月，还需要进一步长期随访的多中心、前瞻性研究来验证本研究的结果。
